# A Study on Improving the Efficacy of Nanoparticle-Based Photothermal Therapy: From Nanoscale to Micron Scale to Millimeter Scale

**DOI:** 10.3390/ma14092407

**Published:** 2021-05-05

**Authors:** Qingyun Jiang, Xinlei Li, Chengping Yin

**Affiliations:** 1 Guangdong Provincial Key Laboratory of Quantum Engineering and Quantum Materials, School of Physics and Telecommunication Engineering, South China Normal University, Guangzhou 510006, China; 2018021884@m.scnu.edu.cn; 2 MOE Key Laboratory of Laser Life Science, Institute of Laser Life Science, College of Biophotonics, South China Normal University, Guangzhou 510631, China; xlli@scnu.edu.cn; 3 Guangdong Provincial Key Laboratory of Laser Life Science, College of Biophotonics, South China Normal University, Guangzhou 510631, China; 4 Guangzhou Key Laboratory of Spectral Analysis and Functional Probes, College of Biophotonics, South China Normal University, Guangzhou 510631, China

**Keywords:** photothermal therapy, nanoparticles size, irradiation shape, temperature distribution, overheating

## Abstract

Photothermal therapy based on nanoparticles is a promising method for cancer treatment. However, there are still many limits in practical application. During photothermal therapy, improving therapeutic effect is contradictory to reducing overheating in healthy tissues. We should make the temperature distribution more uniform and reduce the damage of healthy tissue caused by overheating. In the present work, we develop a simple computational method to analyze the temperature distribution during photothermal therapy at three levels (nanoscale, micron scale, and millimeter scale), and investigate the effects of nanoparticle size, volume fraction, light intensity, and irradiation shape on temperature distribution. We find that it is difficult to achieve good therapeutic effect just by adjusting the volume fraction of nanoparticles and light intensity. To achieve good therapeutic effect, we propose a new irradiation shape, spot array light. This method can achieve a better temperature distribution by easily regulating the positions of spots for the tumor with a large aspect ratio or a small one. In addition, the method of irradiation with spot array light can better reduce the overheating at the bottom and top of the tumor than the full-coverage light or others such as ring light. This theoretical work presents a simple method to investigate the effects of irradiation shape on therapy and provides a far more controlled way to improve the efficacy of photothermal therapy.

## 1. Introduction

Photothermal therapy is a promising alternative therapy to traditional cancer therapy due to a better treatment effect, less pain, and fewer side effects [1,2,3,4]. The principle is to inject the material with high photothermal conversion efficiency into the body and gather them near tumor tissue by targeted recognition technology. Under the irradiation of an external light source, the material converts light energy into heat energy, which causes the tissue temperature increase to kill the cancer cells [5,6,7,8,9]. Since light usually irradiates the entire top surface of the tumor in photothermal therapy, the method often leads to the uneven temperature distribution of tumor and overheating, which results in incomplete treatment of tumor and damage of healthy tissue. These problems limit the further development and application of photothermal therapy. 

Researchers have made efforts basically to study the surface cooling and heating strategy to overcome the uneven temperature distribution and overheating. Dombrovsky et al. [10] found that the period heating strategy can prevent the overheating of tissue surface. However, the proposed method cannot solve the problem of uneven temperature distribution. Ren et al. [11] used the Monte Carlo method and Beer’s law to calculate the heat generation of nanoparticles irradiated by laser. They found that irradiation with a smaller radius than tumor radius can achieve better therapeutic results. Nevertheless, this method is suitable for only tumors with a large aspect ratio, and not for tumors with a small aspect ratio. Furthermore, Wang et al. [12] found that irradiation with ring light can also bring about a better therapeutic effect, but how to regulate the shape of the ring was not studied. Changing the shape of light irradiation provides a way to improve the therapeutic effect. However, there are still some problems to be solved for irradiation with a smaller light radius and ring light, such as further reducing overheating and difficulty in adjusting the shape for optimal temperature distribution. 

In this work, we develop a computational method based on the heat conduction equation to investigate the temperature distribution at three levels (nanoscale, micron scale, and millimeter scale). We use this method to analyze the effects of volume fraction, light intensity, and irradiation shape on temperature distribution. We propose a method of irradiation with spot array light. This method can obtain better temperature distributions of tumors with both large and small aspect ratios. More importantly, it is easy to regulate the irradiation shape for obtaining better tumor temperature distributions, and the irradiation with this shape can better reduce the damage to healthy tissue caused by overheating. 

## 2. Theory and Methods 

The process of photothermal therapy consists of injecting the photothermal conversion material into the body, and the material converts light energy into heat energy under external light source irradiation, which causes a temperature increase in tissue to kill the cancer cells. The essence of the tumor temperature increase is the photothermal conversion of nanoparticles and heat transfer from nanoparticles to cells and then to tumor tissue. Therefore, we can divide the process into three levels by scale: (i) nanoscale (temperature distribution caused by the photothermal conversion of nanoparticles), (ii) micron scale (temperature distribution caused by the heat transfer from nanoparticles to cells), and (iii) millimeter scale (temperature distribution of tissue and the surrounding environment). 

### 2.1. Temperature Distribution Caused by the Photothermal Conversion of Nanoparticles (Nanoscale)

**Figure 1:**
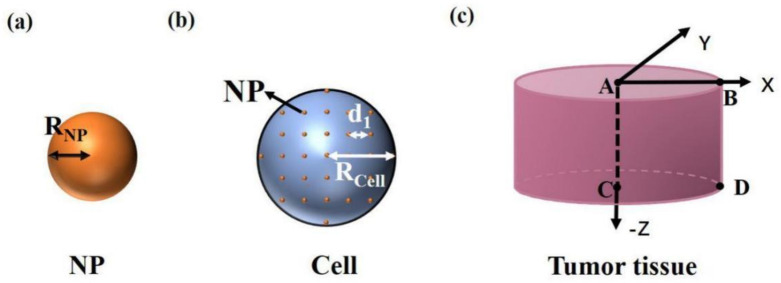
Simplified physical model of (**a**) a nanoparticle, (**b**) a cell, and (**c**) tumor tissue.

Sphere nanoparticles (SNPs) have the simplest shape and are easy to fabricate and widely used in photothermal therapy [6,13]. Under irradiation, SNPs can couple with light within picoseconds. Then, heat transfer from hot SNPs to the surrounding medium takes place, and finally thermal equilibrium is established in nanoseconds [5]. The thermal equilibrium results in a temperature increase in the nanoparticles and the surrounding environment, which is related to the light intensity, absorption cross section of SNP, and density of SNPs in the environment. In detail, high light intensity, large absorption cross section, and high density of SNPs mean a strong increase in temperature. Taking an isolated sphere nanoparticle with radius $R_{NP}$ as an example, as shown in Figure 1a, temperature distribution can be obtained by the heat conduction equation [14,15,16]. The temperature distribution after equilibrium can be given as

(1) $$\Delta T_{np}=\frac{\sigma_{\mathrm{abs}}I}{4\pi \kappa }\frac{1}{r_{}}\left\{\begin{array}{c} r=R_{NP},|\vec{r}|<R_{NP}\\ r=|\vec{r}|,|\vec{r}|\geq R_{NP} \end{array}\right.$$

where κ is the thermal conductivity of the medium, $\sigma_{\mathrm{abs}}$ is the absorption cross section of a single sphere nanoparticle, which is estimated by Mie theory [14,16,17,18], and $\vec{r}$ is the position vector of a point in the irradiation region. At the point outside the particle ($|\vec{r}|\geq R_{NP}$), r is equal to the distance from the point to the center of the nanoparticles ($r=|\vec{r}|$). At the point inside the particle ($|\vec{r}|<R_{NP}$), r is equal to the radius of the nanoparticle ($r=R_{NP}$). *I* is the light intensity. According to Beer’s law, the variation in light intensity with depth is very small on the nanoscale. We consider that the light intensity is constant when we calculate the temperature distribution caused by nanoparticles. 

If there are multiple nanoparticles in the irradiation region, the temperature increase is due to the sum of the contributions of all nanoparticles. Therefore, the temperature distribution caused by the photothermal conversion of multiple nanoparticles is as follows:

(2) $$\Delta T_{nps}=\sum_{i=1}^{N_{1}}\frac{\sigma_{abs}I}{4\pi \kappa }\frac{1}{r}\left\{\begin{array}{l} r=R_{NP},|\vec{r}-{\vec{r}}_{i}|<R_{NP}\\ r=|\vec{r}-{\vec{r}}_{i}|,|\vec{r}-{\vec{r}}_{i}|\geq R_{NP} \end{array}\right.$$

where $N_{1}$ is the number of SNPs, $\vec{r}$ is the position vector of a point in irradiation region, and ${\vec{r}}_{i}$ is the position vector at the *i*th SNP center. If the point is located outside the *i*th nanoparticle ($|\vec{r}-{\vec{r}}_{i}|\geq R_{NP}$), r is equal to the distance from the point to the center of the *i*th nanoparticle ($r=|\vec{r}-{\vec{r}}_{i}|$). If the point is located inside the *i*th particle ($|\vec{r}-{\vec{r}}_{i}|<R_{NP}$), r is equal to the radius of the nanoparticle ($r=R_{NP}$).

### 2.2. Temperature Distribution Caused by the Heat Transfer from Nanoparticles to Cells (Micron Scale)

During photothermal therapy, the modification of nanoparticles by targeted materials can make nanoparticles directional in the cancer cells, and there are very few nanoparticles outside the cells [19]. The common targeted coatings include polyethylene glycol (PEG), transactivator of transcription (TAT) peptide, Arg-Gly-Asp (RGD) peptide, etc. [5]. So we only consider the heat production of particles in the cell. A cell with radius $R_{cell}$ is shown in Figure 1b. We consider that SNPs are uniformly distributed in the cells after being injected into the tissue. *d*_1_ is the distance between particles. The heat production of nanoparticles is much larger than that of the surrounding environment in cells because of the high photothermal conversion efficiency of nanoparticles [20,21]. So, only the heat production of nanoparticles is considered. The temperature distribution caused by the heat transfer from SNPs to cells is affected by each SNP in the cell, which is as follows:

(3) $$\Delta T_{cell}=\sum_{i=1}^{N_{np}}\frac{\sigma_{abs}I}{4\pi \kappa }\frac{1}{r}\left\{\begin{array}{l} r=R_{NP},|\vec{r}-{\vec{r}}_{i}|<R_{NP}\\ r=|\vec{r}-{\vec{r}}_{i}|,|\vec{r}-{\vec{r}}_{i}|\geq R_{NP} \end{array}\right.$$

where $N_{np}$ is the number of nanoparticles in a cell. According to Beer’s law, the variation of light intensity with depth is very small on the micron scale. We still consider that the light intensity is constant when we calculate the temperature distribution caused by cells, because the cell size is tens of microns. 

When there are multiple cells in the irradiation region, the temperature distribution is affected by each particle in each cell, which can be obtained by

(4) $$\Delta T_{cells}=\sum_{j=1}^{N_{2}}\sum_{i=1}^{N_{np}}\frac{\sigma_{abs}I}{4\pi \kappa }\frac{1}{r}\left\{\begin{array}{l} r=R_{NP},|\vec{r}-{\vec{r}}_{ij}|<R_{NP}\\ r=|\vec{r}-{\vec{r}}_{ij}|,|\vec{r}-{\vec{r}}_{ij}|\geq R_{NP} \end{array}\right.$$

where $N_{2}$ is the number of cells, $\vec{r}$ is the position vector of a point in the irradiation region, and ${\vec{r}}_{ij}$ is the position vector of the *i*th nanoparticle in the *j*th cell. Because the number of nanoparticles ($N_{2}\times N_{np}$) in the tissue is very large, the calculation will become very complex. It will take a long time to simulate the temperature field by using software. We find that the calculation can be simplified by taking one cell as a whole. In this case, the temperature distribution of the cells and the surrounding environment can be approximately expressed as

(5) $$\Delta T_{cells}=\sum_{j=1}^{N_{2}}\frac{N_{np}\cdot \sigma_{abs}I}{4\pi \kappa }\frac{1}{r}\left\{\begin{array}{l} r=R_{cell},|\vec{r}-{\vec{r}}_{j}|<R_{cell}\\ r=|\vec{r}-{\vec{r}}_{j}|,|\vec{r}-{\vec{r}}_{j}|\geq R_{cell} \end{array}\right.$$

where $N_{2}$ is the number of cells, $R_{cell}$ is the radius of the cell, $\vec{r}$ is the position vector of a point in the irradiation region, and ${\vec{r}}_{j}$ is the position vector at the *j*th cell center. If the point is located outside the *j*th cell ($|\vec{r}-{\vec{r}}_{j}|\geq R_{\mathrm{cell}}$), r is equal to the distance from the point to the center of the *j*th cell ($r=|\vec{r}-{\vec{r}}_{i}|$). If the point is located inside the *j*th cell ($|\vec{r}-{\vec{r}}_{i}|<R_{cell}$), r is equal to the radius of the cell ($r=R_{cell}$).

By comparing the temperature distribution obtained by the simplified method (Equation (5)) with that obtained by Equation (4), we find that the temperature distribution obtained by the two methods is almost identical outside cells and there is only about 0.1 °C difference inside cells (The comparison is shown in Figure 3). The temperature rise in photothermal therapy exceeds 10 °C [9], so the effect of the difference on temperature distribution inside cells can be negligible. 

### 2.3. Temperature Distribution of Tissue and the Surrounding Environment (Millimeter Scale)

When the cell temperature increases, the tissue temperature also increases due to further heat transfer. A tissue with a radius and depth of 2 mm is shown in Figure 1c. We consider that the cells are of the same size, shape, and uniform distribution in the tissue and the distance between cells is 50 µm. The temperature distribution of the tissue and the surrounding environment can be calculated by

(6) $$\Delta T_{tissue}=\sum_{j=1}^{N_{cell}}\frac{N_{np}\cdot \sigma_{\mathrm{abs}}I}{4\pi \kappa }\frac{1}{r}\left\{\begin{array}{l} r=R_{cell},|\vec{r}-{\vec{r}}_{j}|<R_{cell}\\ r=|\vec{r}-{\vec{r}}_{j}|,|\vec{r}-{\vec{r}}_{j}|\geq R_{cell} \end{array}\right.$$

where $N_{cell}$ is the number of cells in the tissue, and other parameters are similar to Equation (5). The variation of light intensity can be obtained from Beer’s law [11,15,18]:

(7) $$I(z)=I_{0}\cdot e^{-(\mu_{a}+\mu_{s}^{\prime })\cdot z}$$

where $I_{0}$ is the incident light intensity, I(z) is the light intensity at the depth *z*, and $\mu_{a}$ and $\mu_{s}^{\prime }$ represent absorption and scattering coefficients, respectively, which are determined by the absorption and reduced scattering coefficients of both the medium and the nanoparticles ($\mu_{a}=\mu_{a,m}+\mu_{a,n}$, $\mu_{s}^{\prime }=\mu_{s,m}^{\prime }+\mu_{s,n}^{\prime }$) [7]. $\mu_{a,n}$ and $\mu_{s,n}^{\prime }$ are the absorption and reduced scattering coefficients of nanoparticles, respectively, which can be expressed as $\mu_{a,n}=0.75f_{v}Q_{a}/R_{NP}$ and $\mu_{s,n}^{\prime }=0.75f_{v}Q_{s}^{\prime }/R_{NP}$ [22,23]. $f_{v}$ is the volume fraction of nanoparticles. $R_{NP}$ is the radius of nanoparticles. $Q_{a}$ and $Q_{{}_{S}}^{\prime }$ stand for the efficiency factor of absorption and scattering for single particles, calculated by means of Mie theory [17,18].

After the tissue temperature rises, continuous irradiation for a period of time can make cancer cells undergo permanent damage, and the irradiation time is related to the tissue temperature. The Arrhenius equation is commonly used to describe the irreversible heating damage rate of biological tissues [24], which can be expressed as $\Omega (t)=A\exp(-E_{a}/RT)\cdot t$. Here $E_{a}$ and A stand for the activation energy and frequency factor, respectively. R is the gas constant, which equals 8.314 J/(mol·K) [25], T is the temperature of tissue, t is irradiation time, and Ω represents the thermal damage rate. If Ω is larger than 1, the tissue is assumed to have permanent damage. So the irradiation time required for permanent damage of tissue at temperature T is $t\geq 1/[A\cdot \exp(-E_{a}/RT)]$. The higher the temperature, the shorter the light time the tissue needs to get permanent damage. 

## 3. Results and Discussion 

In this paper, we take the typical gold sphere nanoparticle (Au SNP) as an example. The common preparation methods of gold nanoparticles include the Turkevich–Frens method and the Brust–Schiffrin method. The principle of the Turkevich–Frens method is that HAuCl_4_ undergoes a reduction reaction to form gold nanoparticles by using citrate, in which the citrate ion is both reducing agent and sealing agent. When producing large-size gold nanoparticles, the amount of citrate should be reduced to 0.05%. Although citrate ions are not sufficient to make all gold ions undergo a reduction reaction, lower citrate content causes small particles to aggregate into larger particles. The principle of the Brust–Schiffrin method is that HAuCl_4_ undergoes a reduction reaction to form gold nanoparticles by using tetraoctyl ammonium bromide (TOAB) in toluene, in which TOAB is both phase transfer catalyst and stabilizing agent. The radius of Au SNP is selected as 20 nm ($R_{NP}=20\;\mathrm{nm}$), as shown in Figure 1a. The corresponding peak plasma wavelength is about 532 nm, and the absorption cross section can be calculated as 4292.98 nm^2^ by Mie theory ($\sigma_{abs}=4292.98\;{\mathrm{nm}}^{2}$) [17,18,26,27]. The thermal conductivity of tumor with Au SNPs is 0.55W/(K·m) (κ=0.55W/(K⋅m)) [11]. The light intensity is selected as 15 W/cm^2^ ($I=15\;{W/\mathrm{cm}}^{2}$). In this paper, we use Matlab (www.mathworks.com) to simulate the temperature distribution. 

### 3.1. Temperature Distribution 

#### 3.1.1. Temperature Distribution Caused by the Photothermal Conversion of Nanoparticles (Nanoscale) 

**Figure 2:**
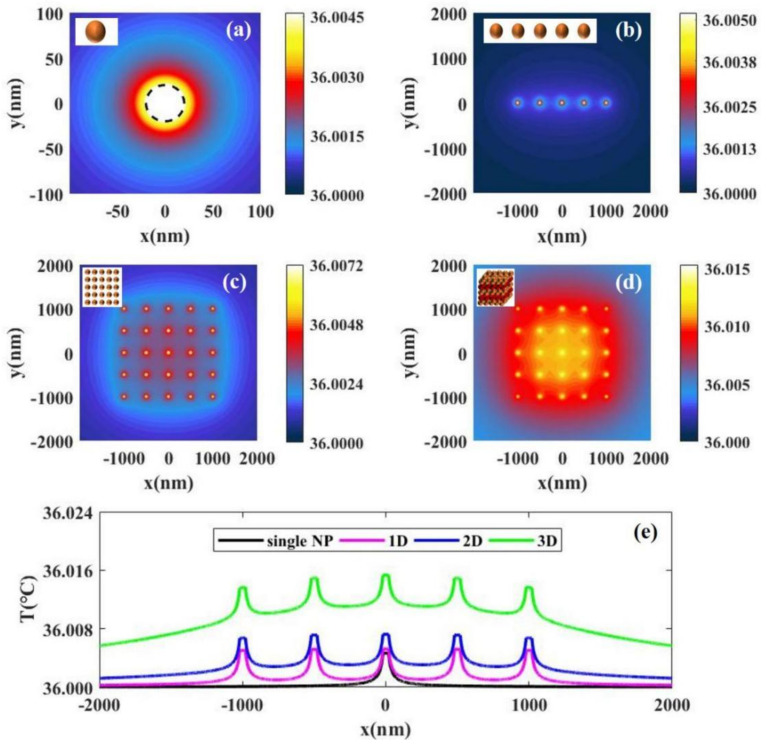
The temperature distributions with laser intensity *I* = 15 W/cm^2^ caused by the photothermal conversion of (**a**) a single nanoparticle, (**b**) 5 nanoparticles uniformly distributed along the x axis, (**c**) 5 × 5 nanoparticles uniformly distributed in the x–y 2D plane, and (**d**) 5 × 5 × 5 nanoparticles uniformly distributed the in x-y-z 3D space. (**e**) A comparison of the temperature distributions caused by the photothermal conversion of single and multiple particles. The distance between the nearest particles is 500 nm.

For nanoscale, we analyze the temperature distribution caused by single and multiple particles. Figure 2a shows the temperature distribution caused by the photothermal conversion of a single Au SNP obtained by Equation (1). The result shows that the temperature inside the particle is the highest and the temperature decreases gradually with increased distance from the particle. Figure 2b–d shows the calculated temperature distributions caused by the photothermal conversion of multiple nanoparticles using Equation (2) in case of one-dimensional ($N_{1}=5$), two-dimensional ($N_{1}=5\times 5$), and three-dimensional ($N_{1}=5\times 5\times 5$). The results show that the higher the number of Au SNPs in the illuminated region, the higher the temperature rise. This is because the temperature rise of the light field is the common action of multiple particles. In addition, Figure 2e shows a comparison of the temperature distributions of single and multiple particles. We can see that the higher the number of particles, the greater the temperature rise, but the temperature rise is always small (ΔT<0.005 ℃). This is because the light intensity that we selected in our calculations is suitable for photothermal therapy and the value of light intensity is small. Interestingly, the conclusion we get from the temperature distributions is similar to that of Baffou et al [14]. Because the light intensity selected in their calculations is much larger than ours, the temperature in their results is higher than ours. 

#### 3.1.2. Temperature Distribution Caused by the Heat Transfer from Nanoparticles to Cells (Micron Scale) 

**Figure 3:**
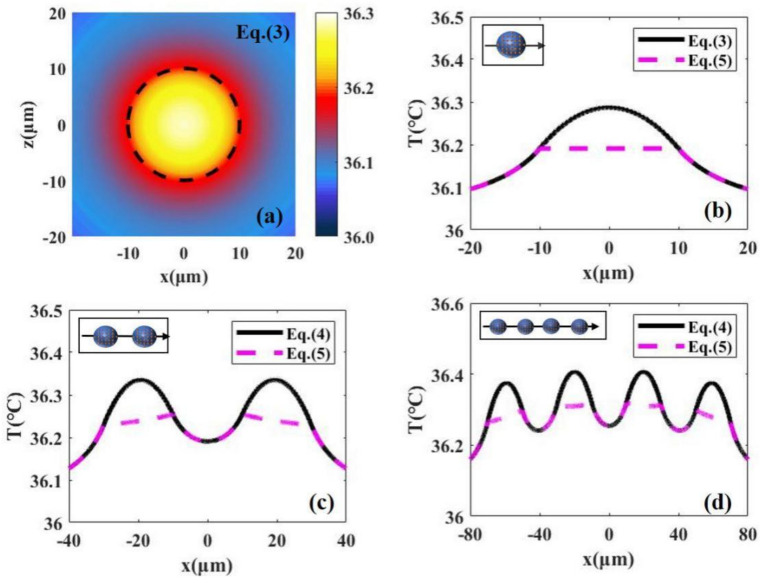
(**a**) The 2D temperature distributions caused by the heat transfer from nanoparticles to a single cell obtained by Equation (2) with laser intensity *I* = 15 W/cm^2^. (**b**) The 1D temperature distribution along the x axis in (**a**). The 1D temperature distributions in the case of two cells and four cells are shown in (**c**) and (**d**). the red dotted lines in (**b**–**d**) represent the calculated results using simplified formulas (Equation (5)).

If nanoparticles enter the cell, we can calculate the temperature distributions of individual or more cells and their surrounding environment by Equations (3) and (4). We consider that the nanoparticles in the cell are uniformly distributed, as shown in Figure 1b. Figure 3a shows the temperature distribution of a single cell by using Equation (3). We find that the temperature of the cell center is the highest, and the temperature around the center gradually decreases, as shown by the black solid line in Figure 3b. Figure 3c,d gives the temperature distributions of two and four cells with their surroundings. We find that the more the cells, the higher the temperature increase, because multiple nanoparticles in cells work together, as shown by the solid lines in Figure 3c,d. In the calculations, we strictly consider the influence of each particle in the system on the temperature distribution using Equation (4). To simplify the calculations, we can consider the cell as a whole and calculate the temperature distributions using Equation (5). The results calculated by Equation (5) are shown by the red dotted lines in Figure 3b–d. We find that the temperature distributions outside cells obtained by Equations (4) and (5) are almost consistent, but the temperature distribution inside cells obtained by Equation (5) is obviously lower than that of Equation (4). When we analyze the temperature distributions of the tumor and the surrounding environment, we focus on the temperature outside the cells. In addition, there are a large number of cells inside the tumor, so the amount of computation is very large if calculated by Equation (4). 

#### 3.1.3. Temperature Distribution of the Tissue and the Surrounding Environment (Millimeter Scale) 

**Figure 4:**
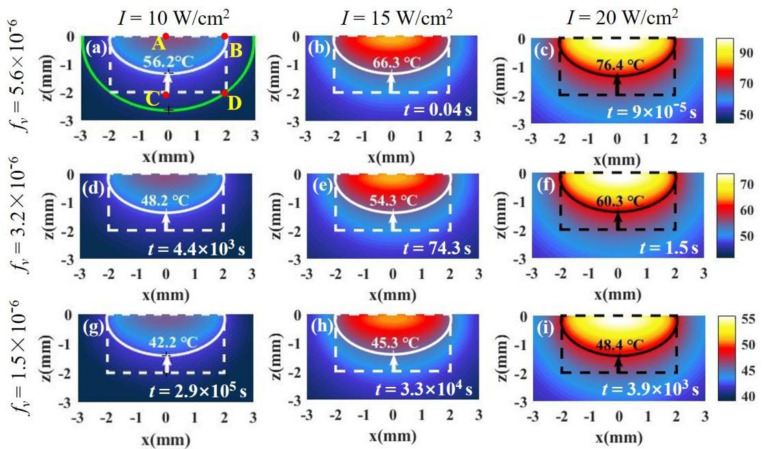
The temperature distributions of tissue and the surrounding environment under different laser intensities and SNP volume fractions. Both tumor depth and radius are 2 mm ($R_{T}=2\;\mathrm{mm}$, z=2 mm). The tumor tissue is in the dotted frame, and healthy tissue is outside of the frame. In (**a**), the white solid line is isotherm through point B, and the green solid line is isotherm through point D. Time *t* is the minimum time required to make tissue undergo permanent damage at the isotherm temperature. The laser intensities and SNP volume fractions in (**a**–**i**) are shown on the top and left of the figure, respectively.

For millimeter scale, we analyze the temperature distribution of a tumor and the surrounding environment. Figure 1c shows a tissue with a cylindrical shape where both radius and depth are 2 mm ($R_{T}=2\;\mathrm{mm}$, z=2 mm). For the millimeter-scale tumor, the light intensity changes dramatically with depth. In this case, we should consider the variation of light intensity with depth, which can be calculated according to Beer’s law (Equation (7)). In our calculations, the absorption and scattering efficiency factor for a single nanoparticle are 3.4 and 0.27, respectively, calculated by means of Mie theory ($Q_{a}=3.4$, $Q_{s}^{\prime }=0.27$) [22,23]. The absorption coefficient and reduced scattering coefficient of the tissue are 32.26/cm and 1.3/cm ($\mu_{a,m}=32.26/\mathrm{cm}$, $\mu_{s,m}^{\prime }=1.3/\mathrm{cm}$), respectively [28]. Figure 4 shows the temperature distributions under different volume fractions of Au SNP and light intensity according to Equation (6) in the case of full-coverage light ($R_{L}\geq R_{T}$). Figure 4a shows the temperature distribution in the case of $f_{v}$ = 5.6 × 10^−6^ and I = 10 W/cm^2^. We can see that the isotherm through point B is above point C, which means that the bottom of the tumor cannot be completely damaged when we do not want to damage healthy tissue. On the other hand, from the isotherm through point D, we can see that it will cause overheating of healthy tissue (see the area indicated by the red arrow) if we want to kill the whole tumor tissue. Therefore, there is a contradiction between improving the therapeutic effect and avoiding overheating of healthy tissue. For the ideal treatment, we hope that the temperature inside the tumor is relatively uniform; that is, the temperature at point B should be close to that at points C and D. 

The reason that the isotherm through point B is above point C is mainly the attenuation of light intensity with depth. We can reduce the volume fraction of particles to weaken the attenuation of light intensity. From Figure 4a,d,g, the lowest point of the isotherm moves down as the volume fraction decreases. We can see that the smaller the volume fraction, the more uniform the temperature distribution, but the temperature decreases at the same time. The results are in agreement with the results calculated by Ren et al. [11]. However, lower temperature means an increase in the treatment time required for irreversible tissue damage. The therapy time can be calculated by the Arrhenius equation [24]. In the figures, the time, *t*, is the minimum time required for irreversible tissue damage. In Figure 4d,g, the treatment times become very long (about 10^3^–10^5^ s) due to the decrease in the nanoparticle volume fraction. We can increase the temperature by increasing the light intensity. By comparing Figure 4a–c, we find that the temperature increases with an increase in light intensity, but the effect of light intensity on the temperature distribution is not obvious. In other words, the position of the isotherm hardly changes with the increase in light intensity. We find that the temperature distribution can be controlled by adjusting the volume fraction, but the temperature distribution is still not ideal. The temperatures at points C and D are still less than that at point B even if the volume fraction is very low. 

### 3.2. Influence of Irradiation Shape on Temperature Distribution 

We know that the temperature at point C is always lower than that at point B from Figure 4. To obtain a better treatment effect, we expect the temperature at point C to be close to that at point B. To investigate whether changing the irradiation shape has a better therapeutic effect, we discuss the effect of irradiation shape on temperature distribution.

#### 3.2.1. Irradiation of Spot Light with a Smaller Radius Than Tissue Radius ($R_{L}<R_{T}$)

**Figure 5:**
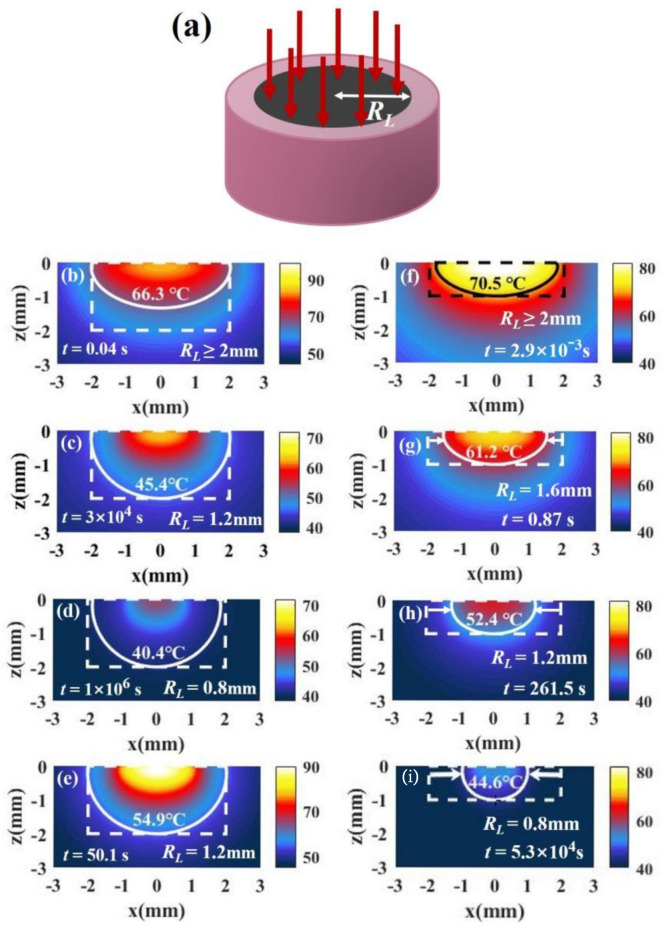
(**a**) Schematic of irradiation of a single spot light with a smaller radius than tissue radius ($R_{L}<R_{T}$). (**b**–**d**) The temperature distributions of a tumor and the surroundings irradiated with *I* = 15 W/cm^2^ and under different irradiation radii for z/RT = 1 ($R_{T}=2\;\mathrm{mm},$ z=2 mm). (**e**) The temperature distribution in case of *R_L_* = 1.2 mm and *I* = 30 W/cm^2^. (**f**–**i**) The temperature distributions with *I* = 15 W/cm^2^ and under different irradiation radii in the case of z/RT = 0.5 ($R_{T}=2\;\mathrm{mm}$, z=1 mm). The volume fraction is 5.6 × 10^−6^ (*f_v_ =* 5.6 × 10^−6^).

The method of irradiation of spot light with a smaller radius can concentrate light energy in the middle of the tissue, and then the temperature on the side will decrease, which can make the temperature at point B and point C closer. The laser irradiation shape is shown in Figure 5a. Figure 5c shows the temperature distribution with $R_{L}=1.2\;\mathrm{mm}$. We find that the temperatures of points B and C are very close, which indicates that the temperature distribution is better than that under irradiation with full-coverage light in Figure 5b. However, the irradiation radius cannot be made too small, because the temperature at the bottom will exceed that at the side when the radius is too small, as shown in Figure 5d. The results are in good agreement with those of Ren et al. [11], who discovered this irradiation strategy. In addition, although the use of irradiation of spot light with a smaller radius can bring about a better temperature distribution, it causes the temperature to decrease (the time required for treatment becomes large). In this case, we can increase the temperature by increasing the light intensity while the temperature distribution hardly changes, as shown in Figure 5e.

Although irradiation of spot light with a smaller radius can improve the therapeutic effect, it may not be good for tumor tissues with a small aspect ratio. We changed the geometry of the tissue to $R_{T}=2\;\mathrm{mm}$ and z=1 mm. For the tumor with a small aspect ratio, the temperature at point C is higher than that at point B under irradiation with full-coverage light, as shown in Figure 5f. If the irradiation radius is reduced, the temperature difference between points B and C becomes larger, as shown in Figure 5g–i. Therefore, reducing the light radius is not useful for improving the temperature distributions of tumors with a small tumor aspect ratio. 

#### 3.2.2. Irradiation with Ring Light

**Figure 6:**
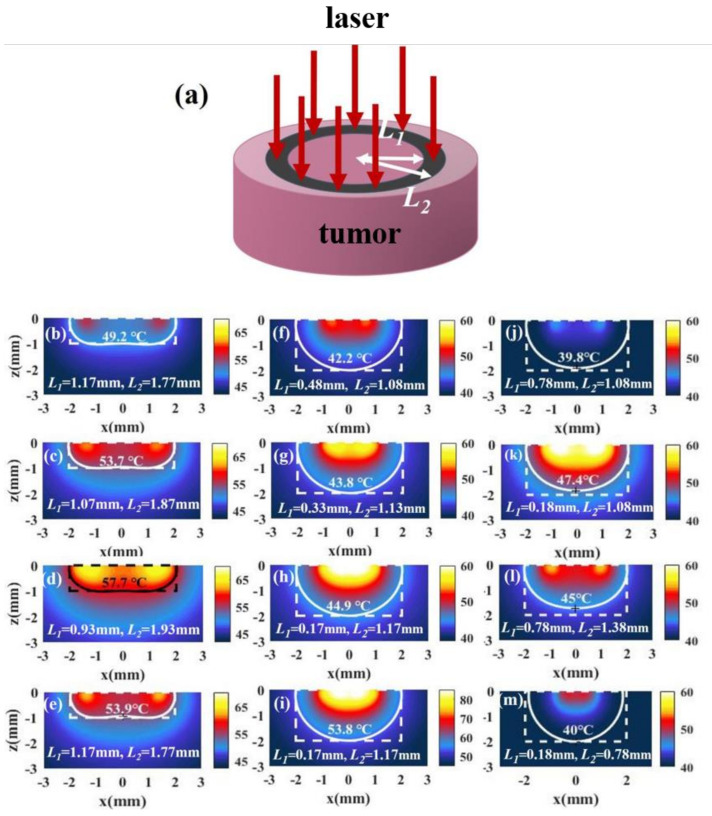
(**a**) Schematic of irradiation with ring light. (**b**–**d**) The temperature distributions of a tumor and the surroundings with *I* = 15 W/cm^2^ and under different irradiation inner radius *L*_1_ and outer radius *L*_2_ in the case of z/RT = 0.5 ($R_{T}=2\;\mathrm{mm}$, z=1 mm). (**e**) The temperature distribution in the case of *L*_1_ *=* 1.17 mm and *L*_2_ *=* 1.77 mm with *I* = 30 W/cm^2^. (**f**–**h**,**j**–**m**) The temperature distribution of a tumor and the surroundings with *I* = 15 W/cm^2^ and different irradiation inner radius *L*_1_ and outer radius *L*_2_ in the case of z/RT = 1 ($R_{T}=2\;\mathrm{mm}$, z=2 mm). (**i**) The temperature distribution in the case of *L*_1_ *=* 0.17 mm and *L*_2_ *=* 1.17 mm with *I* = 30 W/cm^2^.

For tumors with a small aspect ratio, we find that the temperature distributions under irradiation with full-coverage light and irradiation with spot light with a smaller radius are not good because the temperature at the side (point B) is lower than that at the bottom (point C), as shown in Figure 5f–i. To reduce the temperature difference between points B and C, the light should be close to the edge properly. When we use ring light to irradiate the tumor, we can make the light close to the edge of the tissue by adjusting the position of the ring light. Figure 6a shows the laser irradiation strategy of ring light. We find that better temperature distributions can be obtained by using ring light for a tumor with a small aspect ratio, as shown in Figure 6b–d. This method is also effective for a tumor with a large aspect ratio, as shown in Figure 6f–h. However, the temperature of the tissue under irradiation with ring light is small; we can increase the temperature without affecting the temperature distributions by increasing the light intensity, as shown in Figure 6e,i. When we change the width of the ring light to obtain the optimal temperature distribution, we find that the regulation of ring light is difficult, because the temperature distribution is sensitive to the inner and outer radius of the ring light. The temperature distributions vary greatly when the changes in *L*_1_ and *L*_2_ are small, as shown in Figure 6j–m.

#### 3.2.3. Irradiation with Spot Array Light 

**Figure 7:**
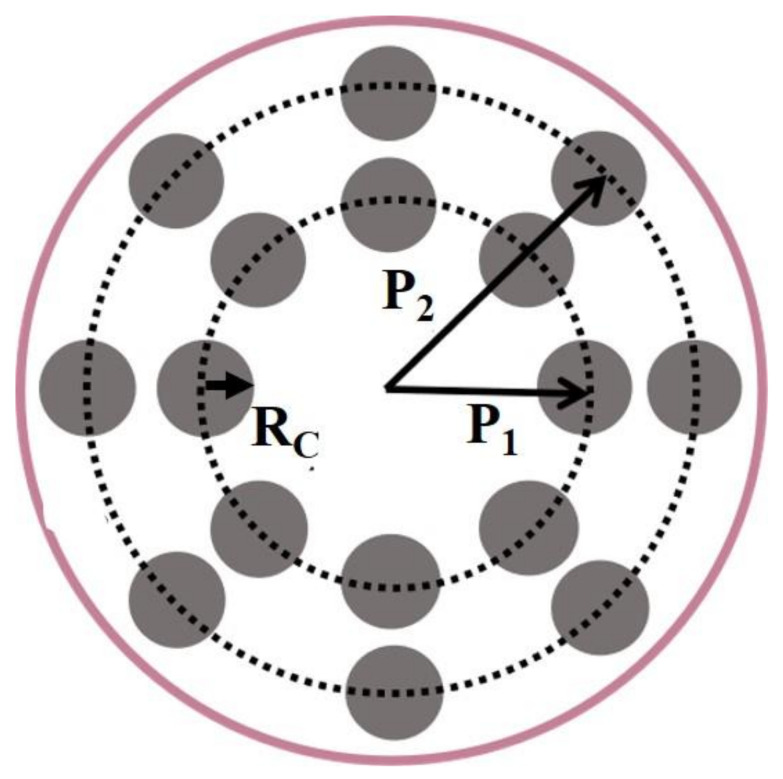
Schematic of spot array light irradiation. $P_{1}$ and $P_{2}$ are the positions of the inner spot and outer spot. In our calculations, the numbers of the inner spot light and the outer spot light are both eight, and the radius of the spot, $R_{C}$ is equal to 0.15 mm.

Although the temperature distribution can be improved by illuminating with ring light, it is difficult to find the optimal shape of the ring because the temperature distribution is sensitive to the position of the ring. To make the irradiation conditions of the optimal temperature distribution easier to regulate, we propose to use irradiation with spot array light, as shown in Figure 7.

**Figure 8:**
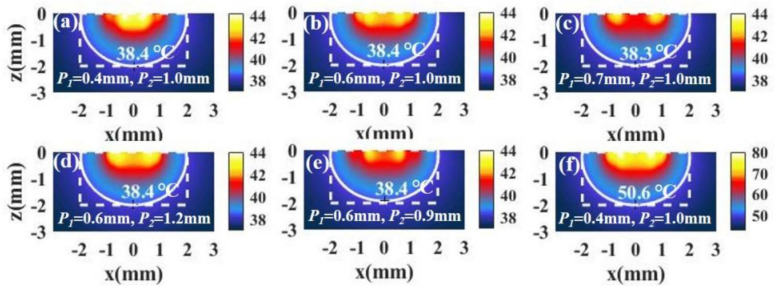
(**a**–**e**) The temperature distributions of a tumor and the surroundings with *I* = 15 W/cm^2^ and the different positions of the inner spot *P*_1_ and the outer spot *P*_2_ in the case of tumor aspect ratio z/RT = 1 ($R_{T}=2\;\mathrm{mm}$, z=2 mm). (**f**) The temperature distribution in the case of *P*_1_ = 0.4 mm and *P*_2_ = 1.0 mm with *I* = 90 W/cm^2^.

Figure 8a shows the temperature distribution in the case of $P_{1}=0.4\;\mathrm{mm}$ and $P_{2}=1.0\;\mathrm{mm}$, where $P_{1}$ and $P_{2}$ represent the positions of the spots in the inner and outer rings, respectively. We find that the irradiation with spot array light can bring about better temperature distributions. Further, we find that the temperature distributions hardly change when the $P_{1}$ and $P_{2}$ change in a small range, as shown in Figure 8b–e. The results indicate that this method is easier to bring about a better temperature distribution than irradiation with ring light. When the temperature is low, we can increase the light intensity to raise the temperature without changing the temperature distribution. Figure 8f shows the temperature distribution after increasing the light intensity. 

**Figure 9:**
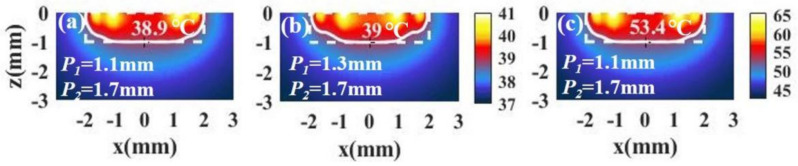
(**a**,**b**) The temperature distributions of a tumor and the surroundings with *I* = 15 × 10^4^ W/cm^2^ and different positions of the inner spot *P*_1_ and the outer spot *P*_2_ in the case of tumor aspect ratio z/RT = 0.5 ($R_{T}=2\;\mathrm{mm}$, z=1 mm). (**c**) The temperature distribution in the case of *P*_1_ = 1.1 mm and *P*_2_ = 1.7 mm with *I* = 90 W/cm^2^.

For tumors with small aspect ratios, a better temperature distribution can also be obtained under irradiation with spot array light. Figure 9a,b shows the temperature distributions of a tumor and the surroundings with different combinations of $P_{1}$ and $P_{2}$. We see that the temperature distribution is also ideal. In addition, we can increase the light intensity to raise the temperature without changing the temperature distribution, as shown in Figure 9c. 

### 3.3. Influence of Nanoparticle Size and Morphology on Temperature Distribution

The size of nanoparticles also affects the therapeutic effect. To fully optimize the photothermal therapy, we discuss the effects of nanoparticle size on temperature distribution. 

**Figure 10:**
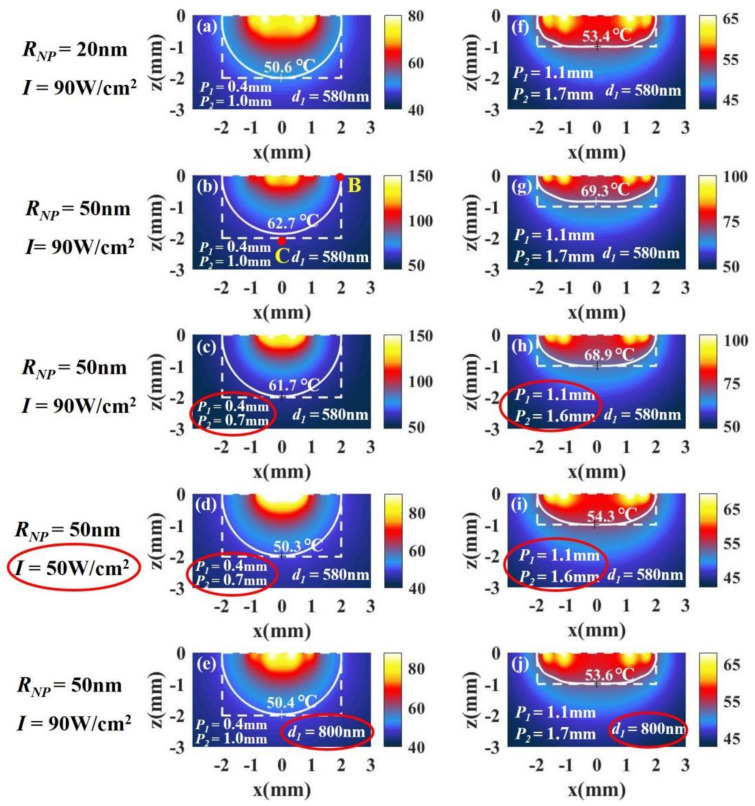
The temperature distributions of a tumor with (**a**–**e**) a large aspect ratio, z/RT = 1 ($R_{T}=2\;\mathrm{mm}$, z=2 mm), and (**h**–**j**) a small aspect ratio, z/RT = 0.5 ($R_{T}=2\;\mathrm{mm}$, z=1 mm). The radius of the nanoparticles is 20 nm in (**a**) and 50 nm in (**b**), and the other parameters used in the calculations in (**a**) and (**b**) are the same. To improve the temperature distribution in (**b**), we can change $P_{1}$ and $P_{2}$ in (**c**). We can reduce the temperature by reducing the light intensity in (**d**). We can also improve the temperature distribution by changing the distance between the particles in (**e**). Similar results can be obtained in the case of small aspect ratios in (**f**–**j**).

When the nanoparticle radius $R_{NP}$ is selected as 50 nm, the temperature distributions in the case of a tumor with a large aspect ratio ($z/R_{T}=1$, $R_{T}=2\;\mathrm{mm}$, z=2 mm) is shown in Figure 10b. We find that the bottom (point C) becomes lower than that at the side (point B), which means that the temperature distribution of tissue with $R_{NP}=50\mathrm{nm}$ is not as good as that with $R_{NP}=20\;\mathrm{nm}$ in Figure 10a (all the parameters except nanoparticle radius in the calculations for (a) and (b) are the same). This is because the volume fraction of the particle with $R_{NP}=50\;\mathrm{nm}$ is larger than that with $R_{NP}=20\;\mathrm{nm}$ when the distance between the particles is the same, so that the light intensity attenuates more strongly with depth. In addition, we find that the temperature of tissue with $R_{NP}=50\;\mathrm{nm}$ is higher than that with $R_{NP}=20\;\mathrm{nm}$. This is because the larger the radius of the particles, the more the light energy absorbed by the particles when the particle radius is below 50 nm in the case of same number of particles. To improve the temperature distribution, we can change the positions of the spots of light in Figure 7. Since the temperature at the bottom (point C) is lower than that at the side (point B) in Figure 10b, the positions of the spots ($P_{1}$ and $P_{2}$) should be appropriately moved inward to concentrate the light energy in the middle of the tissue, as shown in Figure 10c. Although moving the positions of the spots can improve the temperature distribution, the temperature is still much higher than the temperature with $R_{NP}=20\;\mathrm{nm}$. We can reduce the temperature by reducing the light intensity, as shown in Figure 10d. In addition, a good temperature distribution can also be obtained by reducing the volume fraction, i.e., increasing the distance between the particles, $d_{1}$, as shown in Figure 10e. We find that reducing the volume fraction properly can not only improve the temperature distribution but also reduce the temperature. This is because a smaller volume fraction means a decrease in the number of particles. 

For tumors with small aspect ratios ($z/R_{T}=0.5$, $R_{T}=2\;\mathrm{mm}$, z=1 mm), we can get a conclusion similar to that of tumors with large aspect ratios. We find that the temperature distribution of tissue with $R_{NP}=50\;\mathrm{nm}$ is also not as good as that with $R_{NP}=20\mathrm{nm}$, as shown in Figure 10f,g. We can also improve the temperature distribution by moving the positions of the spots and reduce the temperature by reducing the light intensity, as shown in Figure 10h,i. In addition, the temperature distribution can be improved by reducing the volume fraction properly, as shown in Figure 10j. 

Besides the size of nanoparticles, the morphology of nanoparticles is also an important factor affecting the therapeutic effect. Besides spherical gold nanoparticles, nanorods are also commonly applied in photothermal therapy [2,5,21]. The temperature distribution in the case of nanorods can be studied using the presented model, in which the resonance peak and optical parameters (absorption and scattering coefficients) differ from spherical nanoparticles. If we determine the absorption peak and optical parameters of the nanorods, our model can be used to analyze the temperature distribution in the case of nanorods. However, there are two absorbance peaks in gold nanorods, one correlated with the shorter transverse axis around 520 nm and one with the longer longitudinal axis. The longitudinal peak is more sensitive to aspect ratio (length/width), and the longitudinal peak shifts from 640 to 850 nm when the aspect ratio is increased from 1.1 to 4.4 [5]. In calculations, the longitudinal peak is usually chosen as the illumination wavelength, because this wavelength is larger and the skin penetration depth is deeper. Based on our model, we infer that the results in the case of nanorods should be similar to the results in the case of nanospheres. 

### 3.4. Influence of Irradiation Shape on Overheating 

From the above results, we can see that overheating of healthy tissues is inevitable when illuminated with different irradiation shapes, especially the area above the top of the tumor. In the case of tumor tissue with a small aspect ratio, overheating is more obvious.

**Figure 11:**
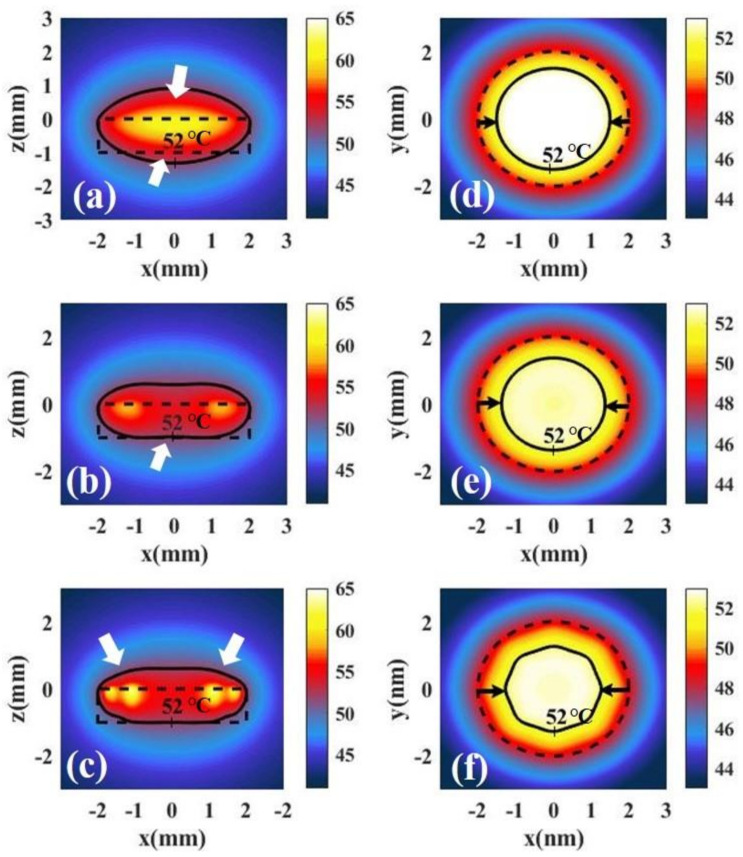
Temperature distributions of tumor tissue at the x–z plane and y = 0 irradiated by different irradiation shapes: (**a**) full-coverage light, (**b**) ring light, and (**c**) spot array light. Temperature distribution of tumor tissue at the x–y, z = 0.5 mm plane irradiated by different irradiation shapes: (**d**) full-coverage light, (**e**) ring light, and (**f**) spot array light.

Figure 11a shows the temperature distribution along the x–z plane (y = 0) under irradiation with full-coverage light. We define healthy tissue over 52 °C as overheating. We find the overheating of healthy tissue at the top and bottom of tumor. Figure 11b shows the temperature distribution under irradiation with ring light. We find that the overheating of healthy tissue at the bottom of tumor can be avoided and the overheating of the top of the tumor can be reduced under irradiation with ring light. Figure 11c shows the temperature distribution under irradiation with spot array light. We find that the overheating at the bottom of the tumor can be avoided, but overheating at the top still exists. To compare the overheating at top of tumor, we calculated the temperature distribution at the x–y, z = 0.5 mm plane under irradiation with three shapes, as shown in Figure 10d–f. We find that the overheating at the top of the tumor under irradiation with ring light is much smaller than that under irradiation with full-coverage light. In addition, we also find that the overheating of healthy tissue at the top of the tumor can be further reduced under irradiation with spot array light. This is because the spot array light makes the light region more dispersed on the top of the tumor. 

Noticeably, during the process of illumination, nanoparticles not only absorb light, but also scatter light. The light scattered by the nanoparticles not only leads to the attenuation of light with depth, but also causes interference between scattered light or between scattered light and incident light, which further affects the absorption of light by particles. In our model, we considered that the light scattered by nanoparticles leads to the attenuation of light with depth. When we use Bill’s law (seen in Equation (7)) to calculate the attenuation of light, the scattering coefficient is one of the most important parameters. However, we ignore the influence of scattered light and interference caused by the scattered light on the absorption of light by particles. This influence should be relatively small. In our model, the absorption coefficient is much larger than the scattering coefficient ($\mu_{a}=39.5/\mathrm{cm}$, $\mu_{s}^{\prime }=1.9/\mathrm{cm}$), so we consider that the effect of scattering on absorption is relatively small. Because the process of scattering and interference is very complex and difficult to be expressed quantitatively, we do not consider the influence of scattering and interference in our model in order to simplify our model. Similar considerations have also been adopted in the literature [9,16], and they yielded results that match the experiments [11,18]. 

## 4. Conclusions 

In this paper, we develop a computational method based on the heat conduction equation to analyze the temperature distribution during photothermal therapy at three levels (nanoscale, micron scale, millimeter scale). We simply analyze or study the effect of volume fraction, light intensity, and irradiation shape on the therapeutic effect by this method. We find that it is difficult to achieve a good therapeutic effect by adjusting the volume fraction of nanoparticles. Furthermore, we improve the therapeutic effect by changing the shape of light. We arrive at some important conclusions.

(1) For tumors with large aspect ratios, reducing the irradiation radius and using ring light are beneficial to the temperature distribution. However, for tumors with small aspect ratios, reducing the irradiation radius cannot get a better temperature distribution, but using ring light can still get a better temperature distribution.
(2) Whether for tumor tissues with a large aspect ratio or small, the method of irradiation with spot array light can achieve a good therapeutic effect. More importantly, this method is easier to regulate for obtaining an ideal temperature distribution than irradiation with ring light.
(3) The smaller nanoparticle radius means better temperature distribution of the tumor. If the nanoparticles are large, we can improve the temperature distribution by increasing the distance between particles or changing the position of illumination.
(4) Overheating under different irradiation shapes is inevitable. The method of irradiation with spot array light can further reduce the overheating at the bottom and the top of a tumor compared to full-coverage light and ring light.

## Data Availability

Data sharing is not applicable to this article.

## References

[1] Ali M.R.K., Wu Y., El-Sayed M.A. (2019). Gold-Nanoparticle-Assisted Plasmonic Photothermal Therapy Advances Toward Clinical Application. J. Phys. Chem. C.

[2] Huang X., Jain P.K., El-Sayed I.H., El-Sayed M.A. (2008). Plasmonic photothermal therapy (PPTT) using gold nanoparticles. Lasers Med. Sci..

[3] Mendes R., Pedrosa P., Lima J.C., Fernandes A.R., Baptista P.V. (2017). Photothermal enhancement of chemotherapy in breast cancer by visible irradiation of Gold Nanoparticles. Sci. Rep..

[4] Doughty A.C.V., Hoover A.R., Layton E., Murray C.K., Howard E.W., Chen W.R. (2019). Nanomaterial Applications in Photothermal Therapy for Cancer. Materials.

[5] Abadeer N.S., Murphy C.J. (2016). Recent Progress in Cancer Thermal Therapy Using Gold Nanoparticles. J. Phys. Chem. C.

[6] Li J., Gu M. (2010). Gold-Nanoparticle-Enhanced Cancer Photothermal Therapy. IEEE J. Sel. Top. Quant. Electro..

[7] Vera J., Bayazitoglu Y. (2009). Gold nanoshell density variation with laser power for induced hyperthermia. Int. J. Heat Mass Tran..

[8] Zakaria H., Abdelaziz W.S., Youssef T. (2016). Effect of size, concentration, and type of spherical gold nanoparticles on heat evolution following laser irradiation using tissue-simulating phantoms. Lasers Med. Sci..

[9] De Oliveira S.A., Borges R., dos Santos Rosa D., de Souza A.C.S., Seabra A.B., Baino F., Marchi J. (2021). Strategies for Cancer Treatment Based on Photonic Nanomedicine. Materials.

[10] Dombrovsky L.A., Timchenko V., Jackson M. (2012). Indirect heating strategy for laser induced hyperthermia: An advanced thermal model. Int. J. Heat Mass Tran..

[11] Ren Y., Qi H., Chen Q., Ruan L. (2017). Thermal dosage investigation for optimal temperature distribution in gold nanoparticle enhanced photothermal therapy. Int. J. Heat Mass Tran..

[12] Wang S.L., Qi H., Ren Y.T., Chen Q., Ruan L.M. (2018). Optimal temperature control of tissue embedded with gold nanoparticles for enhanced thermal therapy based on two-energy equation model. J. Therm. Biol..

[13] Huang X.H., Jain P.K., El-Sayed I.H., El-Sayed M.A. (2007). Gold nanoparticles: Interesting optical properties and recent applications in cancer diagnostic and therapy. Nanomedicine.

[14] Baffou G., Berto P., Bermudez Urena E., Quidant R., Monneret S., Polleux J., Rigneault H. (2013). Photoinduced heating of nanoparticle arrays. ACS Nano.

[15] Qin Z., Bischof J.C. (2012). Thermophysical and biological responses of gold nanoparticle laser heating. Chem. Soc. Rev..

[16] Wang X., He Y., Liu X., Shi L., Zhu J. (2017). Investigation of photothermal heating enabled by plasmonic nanofluids for direct solar steam generation. Solar Energy.

[17] Qin Z., Wang Y., Randrianalisoa J., Raeesi V., Chan W.C., Lipinski W., Bischof J.C. (2016). Quantitative Comparison of Photothermal Heat Generation between Gold Nanospheres and Nanorods. Sci. Rep..

[18] Soni S., Tyagi H., Taylor R.A., Kumar A. (2014). Investigation on nanoparticle distribution for thermal ablation of a tumour subjected to nanoparticle assisted thermal therapy. J. Therm. Biol..

[19] Geng F., Song K., Xing J.Z., Yuan C.Z., Yan S., Yang Q.F., Chen J., Kong B.H. (2011). Thio-glucose bound gold nanoparticles enhance radio-cytotoxic targeting of ovarian cancer. Nanotechnology.

[20] Pissuwan D., Cortie C.H., Valenzuela S.M., Cortie M.B. (2007). Gold nanosphere-antibody conjugates for hyperthermal therapeutic applications. Gold Bull..

[21] Wang Y.C., Black K.C.L., Luehmann H., Li W.Y., Zhang Y., Cai X., Wan D.H., Liu S.Y., Li M., Kim P. (2013). Comparison Study of Gold Nanohexapods, Nanorods, and Nanocages for Photothermal Cancer Treatment. ACS Nano.

[22] Bohren C.F., Huffman D.R. (1983). Absorption and Scattering of Light by Small Particles.

[23] Sroka R., Salas-García I., Lilge L.D., Fanjul-Vélez F., Ortega-Quijano N., Lavín-Castanedo A., Mingo-Ortega P., López-Escobar M., Arce-Diego J.L. (2011). Effect of gold nanoparticles in the local heating of skin tumors induced by phototherapy. Medical Laser Applications and Laser-Tissue Interactions V.

[24] Wright N.T. (2003). On a relationship between the Arrhenius parameters from thermal damage studies. J. Biomech. Eng..

[25] Xu F., Seffen K.A., Lu T.J. (2008). Temperature-dependent mechanical behaviors of skin tissue. IAENG Inter. J. Comput. Sci..

[26] Huang X., El-Sayed M.A. (2010). Gold nanoparticles: Optical properties and implementations in cancer diagnosis and photothermal therapy. J. Adv. Res..

[27] Jain P.K., Lee K.S., El-Sayed I.H., El-Sayed M.A. (2006). Calculated absorption and scattering properties of gold nanoparticles of different size, shape, and composition: Applications in biological imaging and biomedicine. J. Phys. Chem. B.

[28] Bashkatov A.N., Genina E.A., Kochubey V.I., Tuchin V.V. (2005). Optical properties of human skin, subcutaneous and mucous tissues in the wavelength range from 400 to 2000 nm. J. Phys. D Appl. Phys..

